# Novel Prognostic Biomarkers in Gastric Cancer: CGB5, MKNK2, and PAPPA2

**DOI:** 10.3389/fonc.2021.683582

**Published:** 2021-06-15

**Authors:** Min Qin, Zhihai Liang, Heping Qin, Yifang Huo, Qing Wu, Huiying Yang, Guodu Tang

**Affiliations:** ^1^ The First Clinical Affiliated Hospital of Guangxi Medical University, Nanning, China; ^2^ Gastroenterology, Liuzhou People’s Hospital, Liuzhou, China; ^3^ Gastroenterology, Wuzhou Workers’ Hospital, Wuzhou, China; ^4^ The Second Clinical Affiliated Hospital of Guangxi Medical University, Nanning, China

**Keywords:** gastric cancer, WGCNA, tumor immunity, Lasso regression, prognostic biomarkers, immunohistochemistry

## Abstract

**Introduction:**

Gastric cancer is one of the most common malignant tumors of the digestive tract. However, there are no adequate prognostic markers available for this disease. The present study used bioinformatics to identify prognostic markers for gastric cancer that would guide the clinical diagnosis and treatment of this disease.

**Materials and Methods:**

Gene expression data and clinical information of gastric cancer patients along with the gene expression data of 30 healthy samples were downloaded from the TCGA database. The initial screening was performed using the WGCNA method combined with the analysis of differentially expressed genes, which was followed by univariate analysis, multivariate COX regression analysis, and Lasso regression analysis for screening the candidate genes and constructing a prognostic model for gastric cancer. Subsequently, immune cell typing was performed using CIBERSORT to analyze the expression of immune cells in each sample. Finally, we performed laboratory validation of the results of our analyses using immunohistochemical analysis.

**Results:**

After five screenings, it was revealed that only three genes fulfilled all the screening requirements. The survival curves generated by the prognostic model revealed that the survival rate of the patients in the high-risk group was significantly lower compared to the patients in the low-risk group (P-value < 0.001). The immune cell component analysis revealed that the three genes were differentially associated with the corresponding immune cells (P-value < 0.05). The results of immunohistochemistry also support our analysis.

**Conclusion:**

CGB5, MKNK2, and PAPPA2 may be used as novel prognostic biomarkers for gastric cancer.

## Introduction

Stomach cancer ranks fourth among the most common cancerous tumors worldwide, with several factors, such as *H. pylori* infection, diet, and lifestyle, contributing to its development (1). Intestinal epithelialization and the development of atrophic gastritis are reported as the indispensable risk factors for gastric cancer (2). In early gastric cancer, the 5-year survival rate may reach above 95% after treatment with surgery, traditional radiotherapy and chemotherapy, and neoadjuvant therapy (3). It is reported that, at all ages of gastric cancer onset, the presence of metastases at the time of diagnosis is the only factor associated with a poorer prognosis in young adults with gastric cancer (4).

Tumor immunity, a novel approach to cancer treatment, uses immunotherapy to treat the tumors with specific antigens due to mutations in the body cells, facilitating tumor shrinkage (5). Among the immune cells, T cells play an important role in this approach, as the T cells in tumors exhibit extensive dysfunction probably due to the formation of multiple inhibitory signals in the tumor microenvironment (6). Since T cell plays an essential role in the specificity of antigen expression in tumors, it is reported as an important mediator of tumor destruction (7).

With the continuous advancement of bioinformatics, more and more bioinformatics techniques are being used to guide clinical practice and application (8–11). The high prevalence, expensive treatment, and high mortality rate of gastric cancer warrants urgent identification of prognostic biomarkers for gastric cancer for guiding its clinical diagnosis and prognosis. In the present study, a prognostic model of gastric cancer was constructed using precise bioinformatics methods, including the weighted co-expression network analysis (WGCNA), differentially-expressed genes (DEGs) analysis, univariate COX regression analysis, multivariate COX regression analysis, and LASSO regression analysis. Subsequently, CIBERSORT was employed to calculate each sample’s immune cell composition to study the relationship between the sample and the corresponding genes and immune cells.

## Materials and Methods

### Data Download and Initial Processing

Gene expression data and clinical information of the patients with gastric cancer were downloaded from The Cancer Genome Atlas (TCGA) database. The gene expression data were converted to log2 values, and the id names were processed into gene symbols prior to the analysis. The samples with incomplete clinical information were removed. Differentially expressed gene analysis was performed to identify the differentially expressed genes, for the subsequent analysis, using |Log2 Fold Change| > 1 and FDR< 0.05 was the threshold values. All statistical calculations and graphing in the present study were performed in the R software version 4.0.2.

### WGCNA Identification of Significant Modules

A co-expression network was constructed using WGCNA (12), R package, and gene expression matrix. First, a scale-free network was constructed, and the softPower =sft$powerEstimate command R was operated to select the optimal power value automatically. Subsequently, the adjacency matrix was constructed according to the following formula: aij = power (*Sij*, β) = |*Sij*|^β, where aij denotes the adjacency matrix between gene i and gene j, *Sij* denotes the similarity matrix completed by Pearson’s correlations for all gene pairs, and β denotes the soft threshold. Next, the degree of divergence between nodes was calculated, and the adjacency matrix was converted into a TOM matrix. A dynamic shear tree algorithm was then applied to identify the gene networks/modules. Finally, the previously computed module features were compared with the clinical features to analyze the functional modules in the co-expression network.

### GO Enrichment Analysis of Crossover Genes and KEGG Pathway Enrichment Analysis

The clusterProfiler package, the org.H.eg.db package, the enrichplot package, the ggplot2 package, and the GOplot package were used to explore the Gene Ontology (GO) and enriched KEGG pathways of the intersecting genes. The threshold value was set at P < 0.05 and FDR < 0.05, followed by visualization.

### Univariate COX Regression Analysis, LASSO Regression Analysis, and Multivariate COX Regression Analysis

In order to analyze the genes further rigorously, univariate COX regression analysis, multivariate regression analysis, and LASSO regression analysis were performed. The analysis began with the univariate regression analysis, which compared each gene individually with survival time and survival status, and the genes with P-value < 0.05 were selected for the next analysis. Next, the least absolute shrinkage and selection operator (LASSO) regression analysis was performed, which is a sophisticated and advanced method that involves the construction of a penalty function to obtain a further refined model. The genes obtained in this step were analyzed further precisely using the multifactorial Cox regression analysis, with P-value < 0.05 as the significance threshold. Furthermore, each patient’s risk-score was calculated, using which the patients were grouped into high-risk and low-risk groups according to the median risk score.

### Survival Analysis

Here, the patient’s survival curves were analyzed using two different methods to gain insight into the relationship between the high and low gene expressions and the high and low risks predicted by the model and the patient survival. The Kaplan-Meier curves of the two groups were plotted and analyzed in terms of high and low expressions of CGB, MKNK2, and PAPPA2 genes, and accordingly, the patients were divided into high and low expression groups. Subsequently, the Kaplan-Meier curves for these two groups were analyzed in terms of the high and low risk predicted by the constructed model, and accordingly, the patients were divided into high- and low-risk groups.

### Gene Expression Analysis and Principal Component Analysis of High- and Low-Risk Groups

In order to analyze the differences in the genes between the high-risk group and the low-risk group, the differences between the two groups were analyzed using reshape2 and ggpubr packages. Subsequently, the differences in the principal components between the two groups were analyzed using the scatterplot3d package and visualized as a 3D principal component analysis chart.

### ROC Diagnostic Curve and Clinical Correlation Analysis

In order to verify the accuracy of the constructed model, the survival package, the survminer package, and the timeROC package were employed to generate the ROC curves for predicting patient survival at one year, two years, and three years. To further analyze the relationship between the prognostic model and clinical information, we aligned a correlation analysis, in which survival status, survival time, and other clinical information were subjected to multivariate Cox analysis.

### Risk Assessment

In order to validate the accuracy of the constructed prognostic model, the relationship between high and low risk and the survival time was determined for each patient. The patients were ranked according to their risk score [from low to high], and heat maps were plotted for the three genes that were used to construct the model.

### Predicting the Probability of Patient Survival Through Modeling

The rms package was employed to predict and test the risk profile of the constructed model. A calibration chart was prepared to evaluate the accuracy of the constructed model, and a line graph was used to predict patient survival.

### Proportional Assessment of Immune Cell Types and Immune Cell Composition of Model Genes

In order to quantify the immune cell composition of each sample, the proportion of immune cells was evaluated using the CIBERSORT software in the expression matrix of gastric cancer. CIBERSORT is a common tool for characterizing the composition of the immune cells for complex gene expression profiles (13). Here, CIBERSORT was used to identify the composition of immune cells in each sample, with a P-value < 0.05 as the significance threshold. In addition, the composition of immune cells in the individual samples of each gene was determined for the three genes used for constructing the model and correlation analysis.

### Immunohistochemical Analysis

We used pathological tissue sections and paraneoplastic tissue sections of gastric cancer patients who underwent surgical treatment at the First Clinical Affiliated Hospital of Guangxi Medical University for immunohistological studies, and our study was approved by the Ethics Department of the First Clinical Affiliated Hospital of Guangxi Medical University, in accordance with the World Medical Association Declaration of Helsinki. We performed immunohistochemistry on a total of 36 pathological tissue sections for each gene in 6 pairs (gastric cancer and paraneoplastic tissue). CGB5, MKNK2 and PAPPA2 antibodies for immunohistochemical staining were purchased from Abcam (https://www.abcam.cn/, item numbers: ab131170, ab272591 and ab228434). Specimens were removed from paraffin, hydrated, sealed, mixed with anti-CGB5, MKNK2 and PAPPA2, and incubated overnight at 4°C. Subsequently, we performed immunohistochemistry for specific antigen staining on all pathological tissue sections.

## Results

### Data Download and Differential Expression Analysis

**Figure 1:**
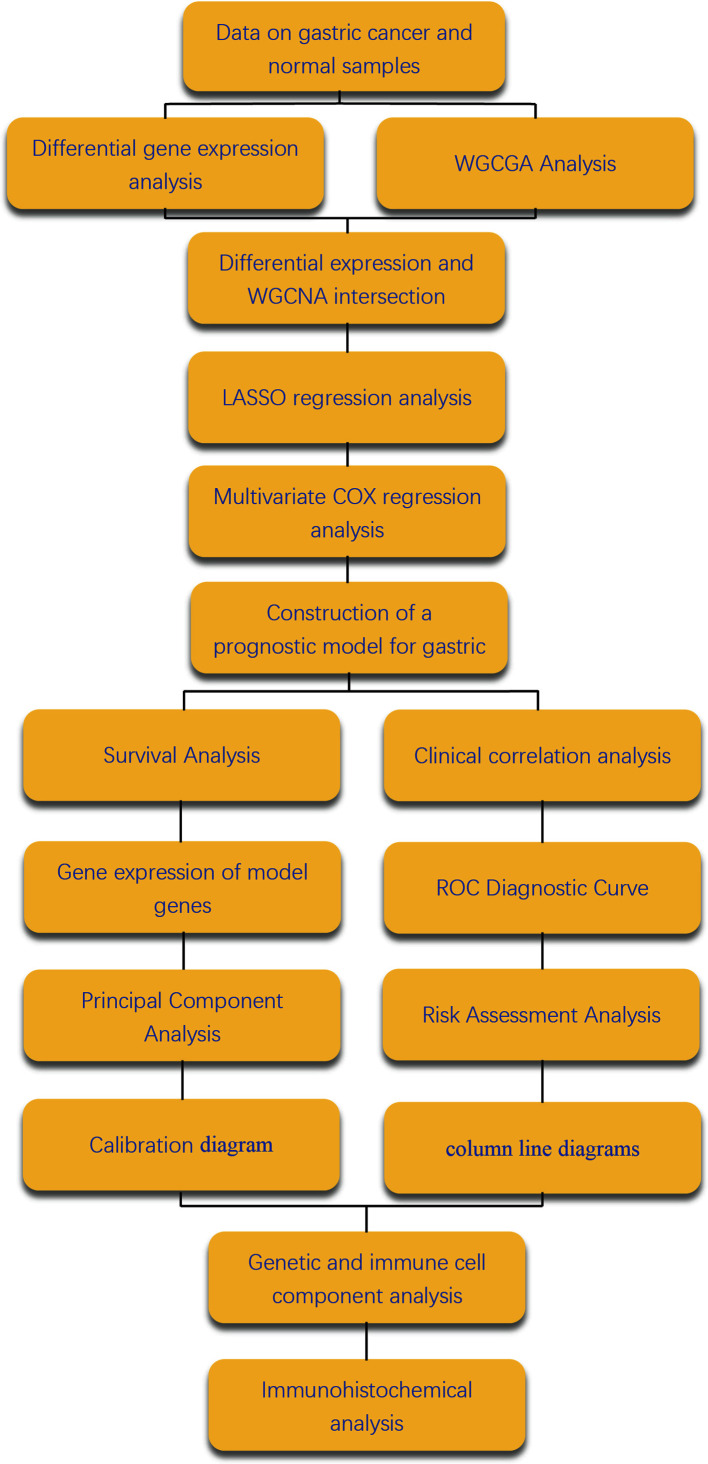
Workflow diagram. **Figure 1** shows the flow of the work done in this study is shown in the figure.

**Figure 2:**
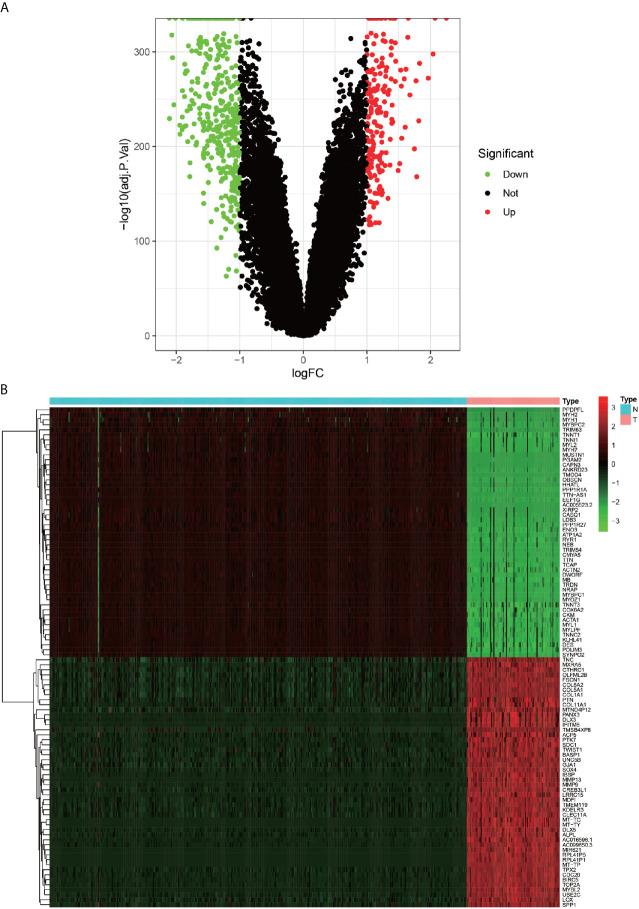
Heat map and volcano map of differentially expressed genes. **(A)** green dots indicate down-regulated differential genes, red dots indicate up-regulated differential genes, and black dots indicate other genes that do not meet the screening criteria. **(B)** Heat map of differentially expressed genes, red squares indicate highly expressed genes.

We have placed the workflow diagram for this study in **Figure 1**. The gene expression profiles of 343 gastric cancer samples and 30 corresponding healthy samples were downloaded from the TCGA database along with the corresponding clinical information data of the gastric cancer samples. A total of 2684 DEGs were selected from a total of 19,597 genes in the expression profile and were visualized as heat maps and volcano maps (**Figures 2A, B**), which revealed 50 up-regulated genes and 50 down-regulated genes that differed significantly from each other in the heat maps.

### WGCNA-Identified Modules With High Relevance to Cancer

**Figure 3:**
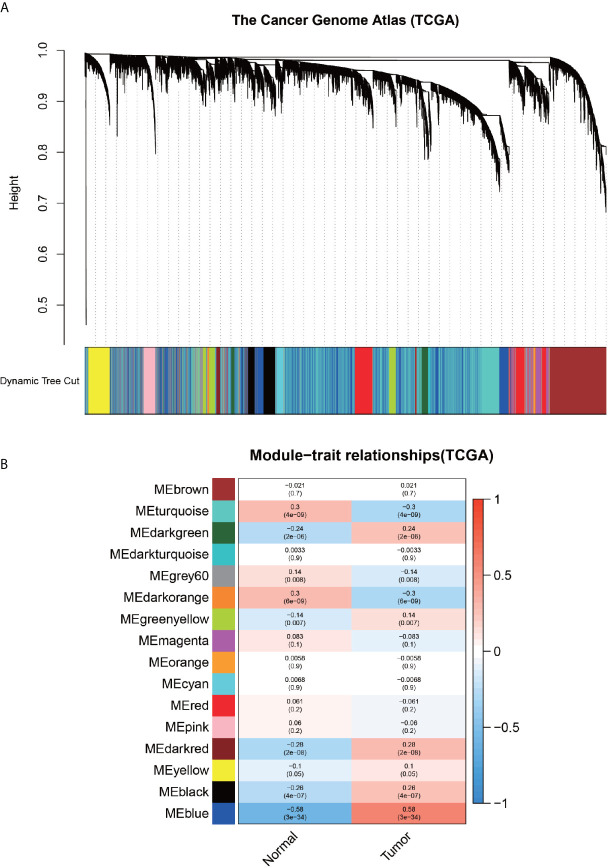
Results of weighted gene co-expression network analysis. **(A)** Diagram, representing the dynamic shearing tree, divides the co-expressed genes into different modules. **(B)**-plot, indicating the Person correlation coefficient of each module with normal and tumor samples.

In order to analyze the differences between the gastric cancer sample data and the healthy sample data in detail, the expression profiles were analyzed using an advanced WGCNA analysis method, the results of which are presented in detail in **Figures 3A, B**. **Figure 3B** depicts that seven modules were positively correlated in tumor samples, and nine modules were positively correlated in the healthy samples. In order to identify the differential genes further precisely, the advantages of two methods were combined to screen the genes of the MEblue module with the highest positive correlation in the tumor through the intersection with DEGs. As depicted in **Figure 5D**, a total of 1012 genes were finally included in the present study.

### GO Enrichment Analysis and KEGG Pathway Enrichment Analysis

**Figure 4:**
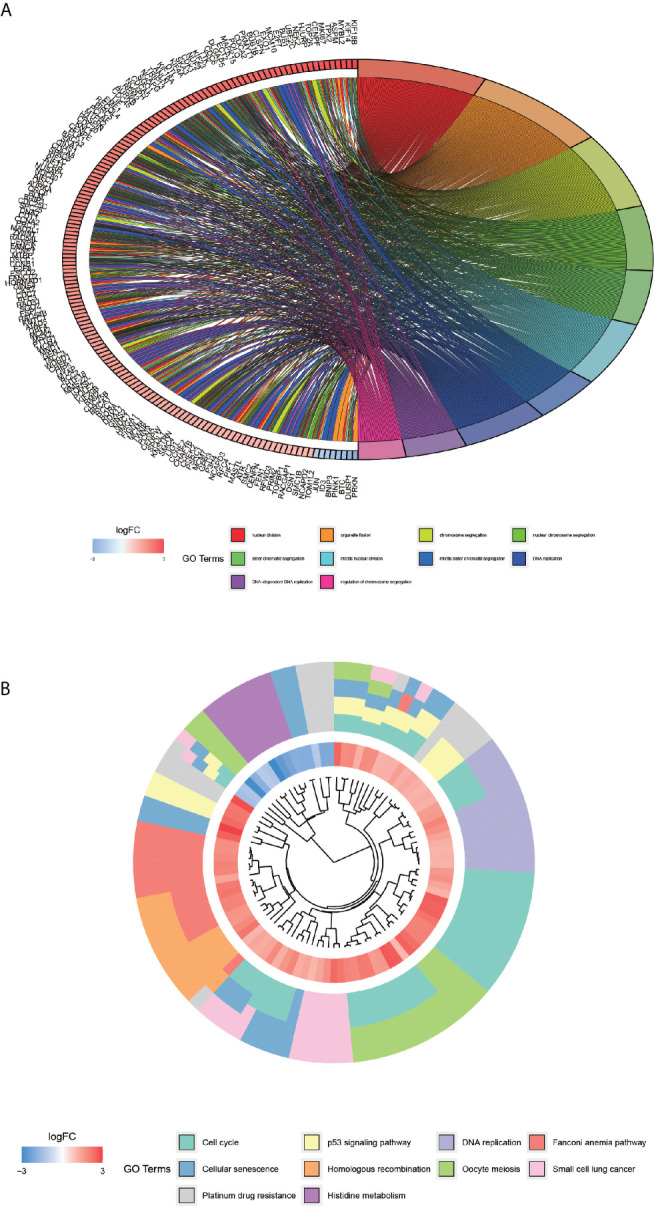
GO enrichment analysis of co-expressed differential genes and KEGG pathway enrichment analysis. Graph **(A)** showing the first 10 GO entries, with each color indicating one entry and the difference in the color of the gene indicating the change in LogFC value. **(B)**-plot, KEGG pathway enrichment analysis. Each color indicates a pathway, and the innermost circle indicates the logFC value.

In order to explore the molecular functions and pathways of the selected 1012 genes, the GO enrichment analysis and KEGG pathway enrichment analysis were performed. The GO enrichment analysis revealed that the GO entries were concentrated mainly in the nuclear division, organelle fission, chromosome segregation, nuclear chromosome segregation, and mitotic nuclear division, etc. (**Figure 4A**), while the KEGG pathway was enriched mainly in the cell cycle, DNA replication, Fanconi anemia pathway, and small cell lung cancer (**Figure 4B**).

### Univariate COX Regression Analysis, Lasso Regression Analysis, and Multivariate COX Regression Analysis

**Figure 5:**
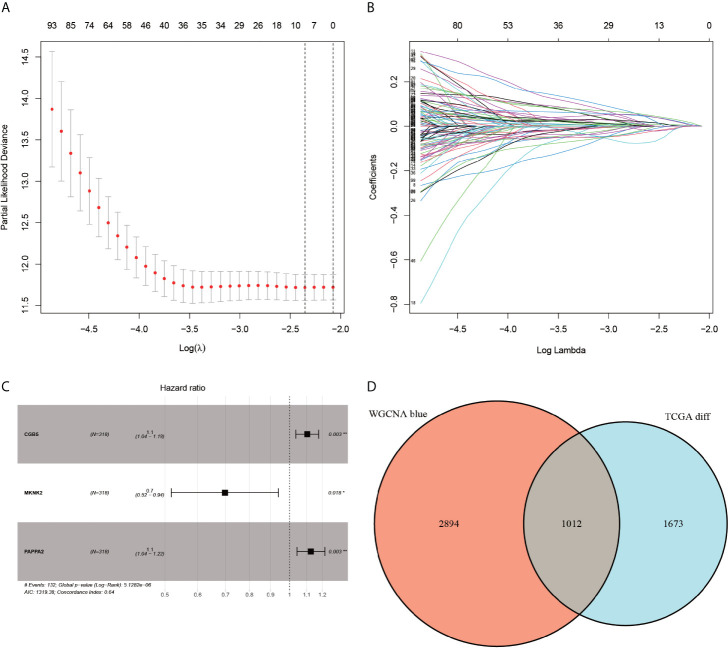
LASSO regression analysis, multi-factor regression analysis and veen plots. Plots **(A, B)** represent the minimum penalty coefficient model constructed using the LASSO regression model. The **(C)**-plot represents the final forest plot obtained for the three genes used to construct the model. The **(D)**-plot represents the Venn diagram of differentially expressed genes with the corresponding modules of WGCNA.

The selected 1012 genes were further analyzed using the univariate COX regression analysis, Lasso regression analysis, and multivariate COX regression analysis. After the univariate COX regression analysis, a total of 138 genes fulfilling the screening requirements were used for the next further analysis, i.e., LASSO regression analysis, which is a further complex method of analysis (**Figures 5A, B**). The ten genes that remained after the filtering with LASSO regression were subjected to multivariate COX regression analysis to compare the survival data of each gene. The only genes that fulfilled all the screening criteria were CGB5, MKNK2, and PAPPA2 (**Figure 5C**). This point marked the completion of the construction of the prognostic model for gastric cancer. Finally, the risk score of each patient was calculated.

### Survival Analysis

**Figure 6:**
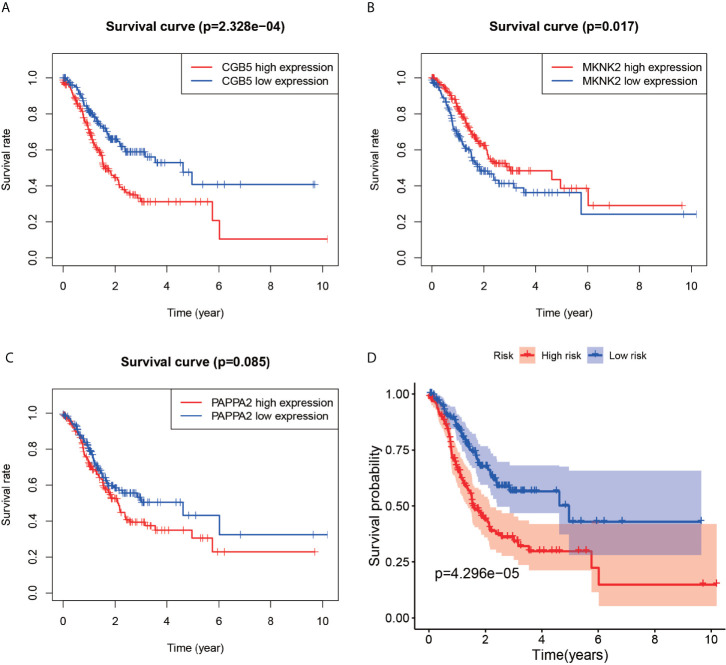
Survival analysis chart. Plots **(A–C)** represent survival analysis plots constructed based on high and low expression of genes. The **(D)**-plot represents the survival analysis plot constructed based on the high and low risk values of the constructed model.

The Kaplan-Meier curves (**Figures 6A–C**) revealed that the survival rate of the patients in the high-expression group of the CGB5 and PAPPA2 genes was lower than that in the low-expression group, with the difference in the survival curve of the CGB5 gene being statistically significant (P-value < 0.001). The Kaplan-Meier curves (**Figure 6D**) plotted for high and low risk revealed that the survival rate of the patients in the high-risk group was much lower than that of the low-risk group (p-value < 0.001).

### Gene Expression Analysis and Principal Component Analysis of High- and Low-Risk Groups

**Figure 7:**
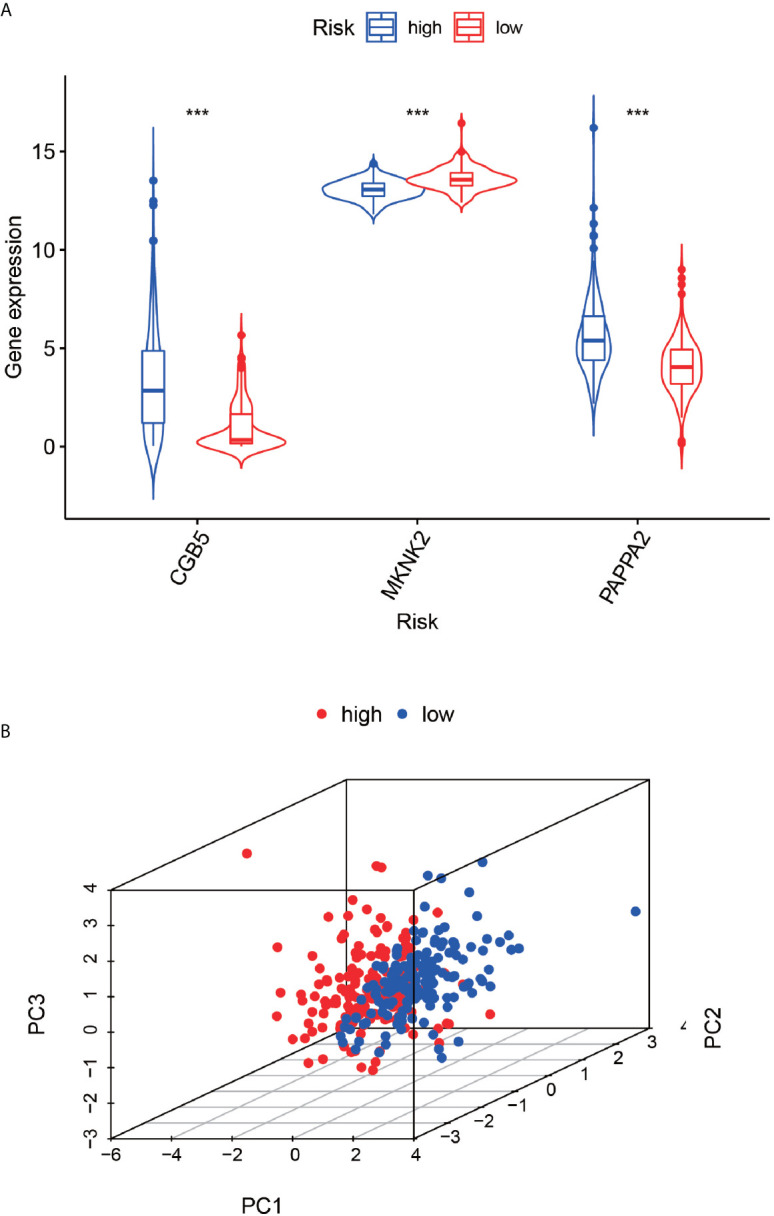
Expression differences between high and low risk groups and principal component analysis. The **(A)**-plot represents the difference in gene expression values of the three genes based on the high and low risk of the constructed model. The **(B)**-plot represents the principal component analysis based on high and low risk, high risk group and low risk group. ***P < 0.001.

The differential violin plots (**Figure 7A**) for the three genes used for constructing the model were analyzed based on the high and low-risk groups. The gene expression in the low-risk groups of CGB5 and PAPPA2 genes was observed to be higher than that in the high-risk group, while the gene expression in the high-risk group of MKNK2 was higher than that in its low-risk group, with all the differences being statistically significant (P-value < 0.001). In the principal components analysis results (**Figure 7B**), the red dots in the high-risk group were concentrated on the left side of the PC1 axis, while the blue dots in the low-risk group were concentrated on the right side of the PC1 axis, with the two groups clearly distinguishable.

### ROC Diagnostic Curve

**Figure 8:**
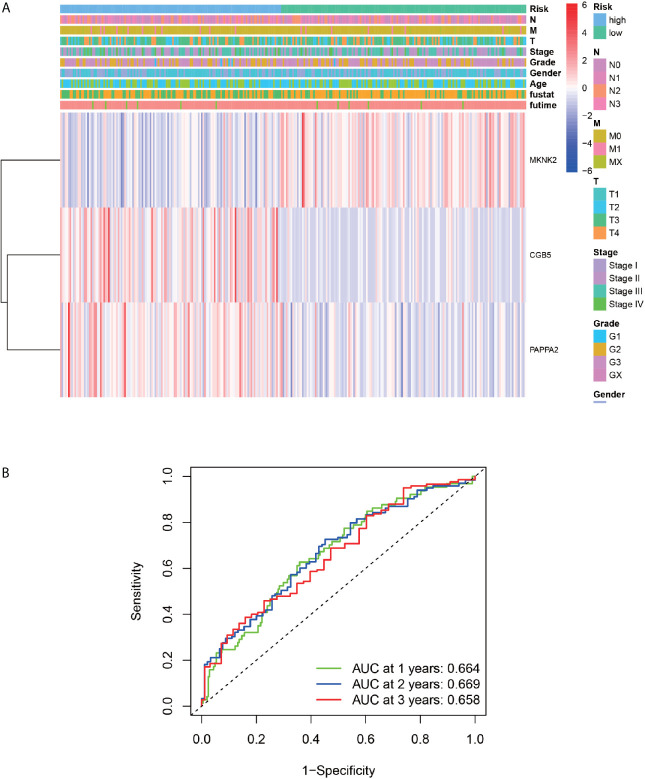
ROC Diagnostic Curve and Clinical Correlation Analysis. **(A)** Represents the relationship between the three genes used to construct the model and the clinical information. **(B)** Represents the AUC values for 1, 2 and 3 years were 0.664, 0.669 and 0.658, respectively.

According to the ROC curve (**Figure 8B**), AUC was 0.664 for one year, 0.669 for two years, and 0.658 for three years, which were all above 0.5, indicating that the constructed model has some feasibility in predicting prognosis. We could find from the multivariate Cox regression analysis (**Figure 8A**) that the expression of MKNK2 was higher in the high-risk group than in the low-risk group, and the expression of CGB5 and PAPPA2 was higher in the low-risk group than in the high-risk group.

### Risk Assessment

**Figure 9:**
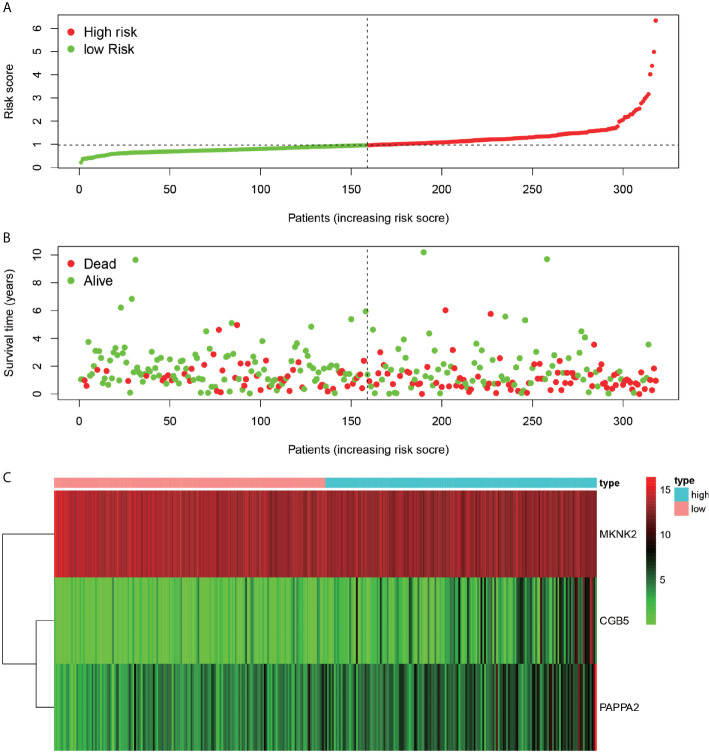
Riskiness assessment. Chart **(A)** represents the ranking of all patients in order from low risk to high-risk line based on the level of risk. Chart **(B)** indicates the survival of individual patients, with red dots indicating death and green dots indicating survival. **(C)**-plots indicate the gene expression of the three genes used to construct the model in each sample.

The gastric cancer patients were ranked low-risk and high-risk cases based on the risk score generated by the constructed model (**Figure 9A**). According to the survival diagram (**Figure 9B**), the patients who died were roughly located in the higher risk-score right-hand region. The risk heat map (**Figure 9C**) depicts that the expression of CGB5 and PAPPA2 increased from the low- to high-risk region, while the expression of MKNK2 decreased from the low- to high-risk region.

### Calibration Charts and Line Graphs

**Figure 10:**
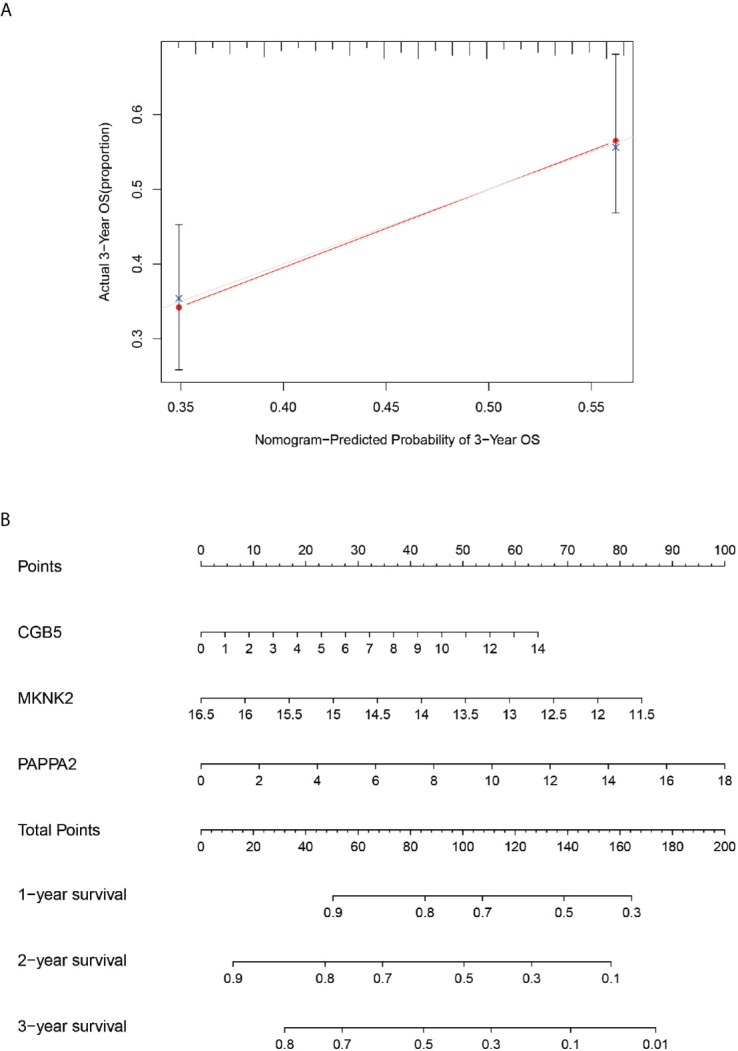
Calibration and column line diagrams. Chart **(A)**, the red line segment indicates actual survival and the gray line segment indicates predicted survival, which almost overlap. The **(B)**-plot represents a column line plot constructed based on three genes and 3-year scores for predicting survival.

The Calibration chart (**Figure 10A**) was prepared to validate the constructed model. The red line (predicted line) in the chart roughly coincides with the actual line (gray line), validating the accuracy of our model. Therefore, the line graph (**Figure 10B**) predicted by the model can be used to predict the 1-year, 2-year, and 3-year survival probabilities for any patient.

### Immunocyte Composition of Samples and Model Genes

**Figure 11:**
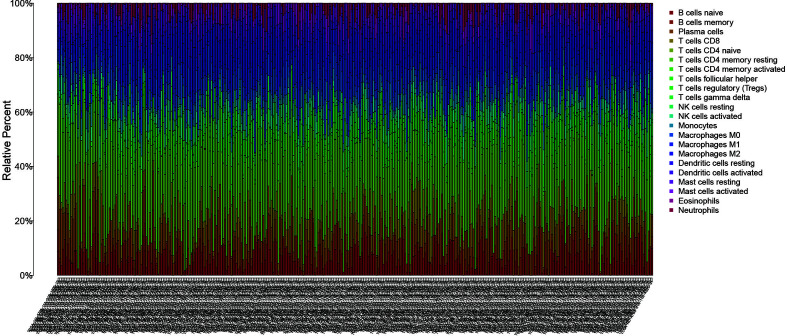
CIBERSORT immunocytometric analysis. The immune cell composition of all samples was analyzed in 22 using CIBERSORT.

**Figure 12:**
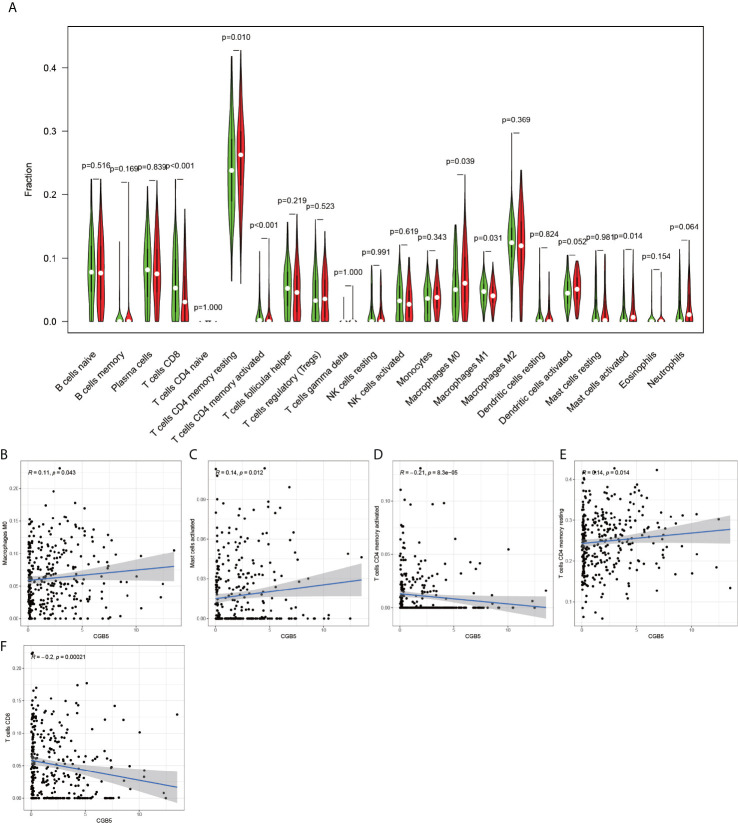
CGB5-based immune cell analysis. The **(A)** graph shows that there are 5 immune cells associated with CGB5 in gastric cancer (P-value < 0.05). Plots **(B, C, E)** indicate that these three immune cells are positively correlated with CGB5. plots **(D, F)** indicate that these two immune cells are negatively correlated with CGB5.

**Figure 13:**
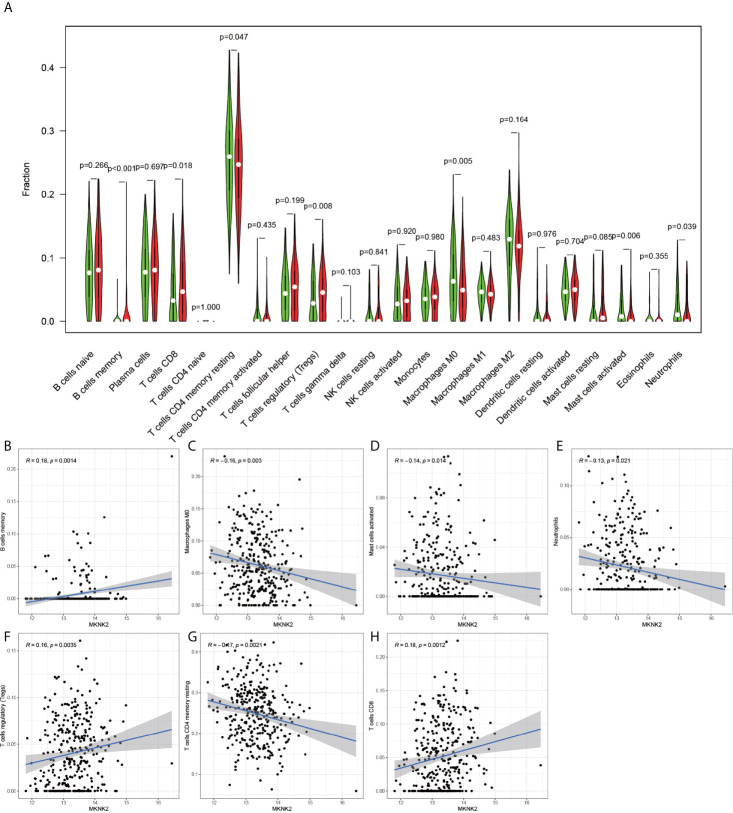
MKNK2-based immune cell analysis. The **(A)** graph shows that there are 7 immune cells associated with MKNK2 in gastric cancer (P-value < 0.05). The graphs **(B, F, H)** indicate that these three immune cells are positively correlated with MKNK2. **(C–E, G)** indicate that these four immune cells are negatively correlated with MKNK2.

**Figure 14:**
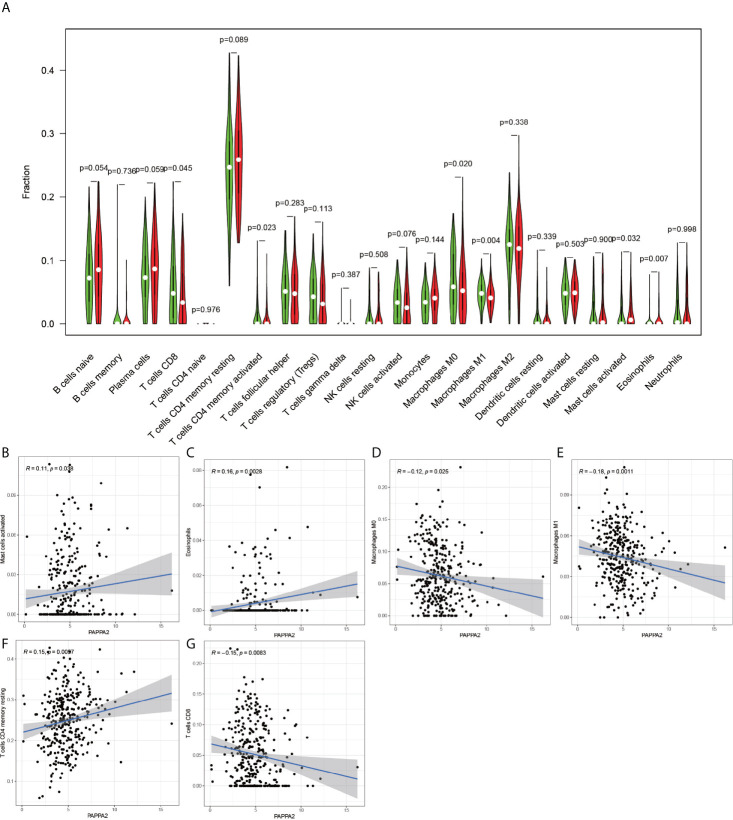
PAPPA2-based immune cell analysis. The **(A)** graph shows that there are 6 immune cells associated with PAPPA2 in gastric cancer (P-value < 0.05). **(B, C, F)** plots indicate that these three immune cells are positively correlated with PAPPA2. **(D, E, G)** plots indicate that these three immune cells are negatively correlated with PAPPA2.

Using the CIBERSORT software, the immune cell composition of all the samples were determined, and the sum of the immune cell composition in each of the 22 samples was 100% (**Figure 11**). The composition of immune cells in the 22 samples for CGB5 gene expression revealed the presence of macrophage M0, macrophage M1, and memory CD4 T cells, as depicted in **Figure 12**. The resting and CD8 T cells demonstrated a significant correlation (P-value < 0.05). In the case of MKNK2 gene expression (**Figure 13**), memory B cells, CD8 T cells, resting memory CD4 T cells, regulatory T cells (Tregs), macrophages M0, activated mast cells, and neutrophils demonstrated a significant correlation (P-value < 0.05). Finally, for PAPPA2 gene expression (**Figure 14**), CD8 T cells activated memory CD4T cells, macrophages M0, macrophages M1, activated mast cells, and eosinophils demonstrated a significant correlation (P-value < 0.05).

### Immunohistochemical Analysis

**Figure 15:**
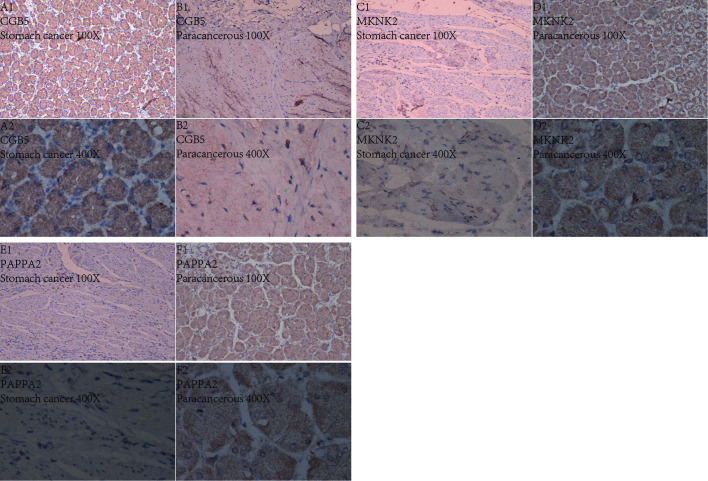
Immunohistochemistry. Figures **(A1–F2)** Show the expression of these three genes in gastric cancer and in paracancerous tissue, respectively. 100X indicates 100x magnification under inverted microscope and 400X indicates 400x magnification under inverted microscope.

After laboratory manipulation, we obtained 36 pathological sections with good staining for immunohistology. We placed all immunohistological images under an inverted microscope for observation and compared the staining differences between gastric cancer specimens and paraneoplastic tissue specimens. We performed immunohistological staining analysis on a total of 36 pathological tissue sections for 6 pairs (gastric cancer and paraneoplastic tissue) for each gene. We analyzed all pathological tissue sections and found that the expression of CGB5 was significantly higher in gastric cancer than in paraneoplastic tissue (**Figures 15A1, B2**). In contrast, the expression of MKNK2 and PAPPA2 was significantly higher in paraneoplastic tissues than in gastric cancer tissues (**Figures 15C1–F2**). This is consistent with the results of our analysis. Thus, the accuracy of our analysis was verified at the laboratory level.

## Discussion

According to the results obtained in the present study, the GO enrichment analysis entries were concentrated mainly in the nuclear division, organelle fission, chromosome segregation, nuclear chromosome segregation, and mitotic nuclear division, while the KEGG pathway was enriched mainly in the cell cycle, DNA replication, Fanconi anemia pathway, and small cell lung cancer. In 2004, it was reported that the DNA damage caused by different factors (e.g., solar radiation) in humans could be managed using cell cycle tests and that the extent of a person’s exposure to these factors and the response of the cells in their body to the DNA damage are critical factors determining whether a person would develop cancer as a consequence (14). Moreover, genomic instability is an important hallmark of cancer. DNA replication is one of the most important cellular processes involved in cancer, and any condition that may lead to DNA damage can produce stress during the replication period along with the corresponding genomic instability, which is one of the main characteristics of cancer and cancerous cells (15).

Chorionic Gonadotropin Subunit Beta 5 (CGB5) is a protein-encoding gene, primarily associated with Invasive Mole and Ectopic Pregnancy. It is reported that in ovarian cancer, CGB5 may activate the LHR signaling pathway and thus appears to promote tumor growth and the formation of angiogenic mimics (16). Recent studies have demonstrated that CGB5, a member of the CGB family, may have an important role in cervical squamous cell carcinoma, pancreatic adenocarcinoma, and rectal adenocarcinoma (17). This is consistent with the findings of our study, in which CGB5 was revealed as a key gene for predicting the prognosis of patients with gastric cancer. In addition, we observed that CGB5 was associated with various immune cells in gastric cancer, presenting positive trends for macrophage M0, activated mast cells and resting memory CD4 T cells (**Figures 12B–D**), negative trends for activated memory CD4 T cell and CD8 T cells (**Figures 12B–D**), and a trend of negative correlation with the activated memory CD4 T cells and CD8 T cells (**Figures 12D, F**). Recent studies have demonstrated that the already apoptotic cells stimulate macrophages M0 and thereby generate the macrophages that promote ovarian cancer migration and proliferation (18). More interestingly, mast cell activation, and resting memory CD4 T cells are reported to be inextricably linked to cancer development (19, 20).

MAPK Interacting Serine/Threonine Kinase 2 (MKNK2) is a protein-coding gene, primarily associated with IL-1 signaling pathway and ERK signaling pathway. Interestingly, the interleukin-1 family is associated with the growth and metastasis of several cancers (21, 22). The present study also found a positive correlation of MKNK2 with memory B cells, regulatory T cells (Tregs), and CD8 T cells in gastric cancer, all of which are reported to be strongly associated with tumors (23–25).

Pappalysin 2 (PAPPA2) is a protein-encoding gene associated with several disorders, including Down’s syndrome and HELLP syndrome. High expression of PAPPA2 is associated with mortality in lung cancer patients (26). Coincidentally, PAPPA2 is also associated with multiple immune cell types in gastric cancer, similar to the present study, in which PAPPA2 exhibited a positive correlation with activated mast cells, eosinophils, and activated memory CD4 T cells, and negative correlation with macrophage M0, macrophage M1, and CD8 T cells. The implementation of immunotherapeutic measures against cancer is being increasingly recognized and endorsed by other researchers as well (27).

In the present study, the constructed prognostic model of gastric cancer was used to calculate the risk score for each patient, according to which the patients were divided into high-risk and low-risk groups. Furthermore, the results of the survival analysis revealed that the 5-year survival rate of the patients in the high-risk group was much lower than those in the low-risk group (P-value < 0.001). The combination of WGCNA and differentially expressed gene analysis used in the present study for pre-screening, which was followed by univariate COX regression analysis, LASSO regression analysis, and multivariate regression analysis, enabled the construction of a highly-accurate prognostic model for gastric cancer. According to the expression of the three genes CGB5, MKNK2, and PAPPA2 used for constructing the constructed model, the patients were divided into high-risk groups and low-risk groups, followed by the calculation of the expression of each gene in both the groups. In addition, a principal component analysis was performed, and the principal components for each patient were plotted in a 3D graph, from which we could clearly distinguish the high-risk and low-risk groups. Moreover, all the AUC values determined from the ROC curves were greater than 0.5, further validating the accuracy of the constructed model. The accuracy of our model is also highlighted by the gentle coinciding of the line predicting the three-year overall survival rate with the line predicting the actual survival rate. Each of the three genes was strongly associated with immune cells, and since the immune process plays an integral role in tumor formation and development, it further validated the accuracy of the constructed model. Finally, we further demonstrated the accuracy of our analysis by performing immunohistochemistry on human gastric cancer tissues as well as paracancerous tissues to analyze the differences in expression of these three genes in cancerous and paracancerous tissues. In conclusion, using sophisticated and precise bioinformatics tools, a prognostic model for gastric cancer was constructed in the present study, and three biomarkers strongly associated with the prognosis of gastric cancer were identified.

As with all research, the present study also had certain limitations. First, the sample size was insufficient. Although 343 gastric cancer samples and 30 healthy samples were included in the present study, it was far from a sufficiently large sample size. Second, the gastric cancer samples were not studied according to each specific type of cancer.

## Conclusion

The CGB5, MKNK2, and PAPPA2 genes may serve as important biomarkers for predicting the prognosis of gastric cancer.

## Data Availability Statement

Publicly available datasets were analyzed in this study. This data can be found here: The datasets supporting the conclusions of this article are available in The Cancer Genome Atlas Program (TCGA, https://www.cancer.gov/about-nci/organization/ccg/research/structural-genomics/tcga).

## Ethics Statement 

The studies involving human participants were reviewed and approved by Department of Medicine, The First Clinical Hospital of Guangxi Medical University. Written informed consent for participation was not required for this study in accordance with the national legislation and the institutional requirements.

## Author Contributions

MQ and GT designed the study. ZL, HQ and YH analyze the data. QW and HY digital visualization. MQ wrote and revised the manuscript. MQ and GT revised the manuscript. All authors contributed to the article and approved the submitted version.

## Funding

This study was sponsored by the Health and Health Commission of Guangxi Zhuang Autonomous Region with self-funding, No. Z20200199.

## Conflict of Interest

The authors declare that the research was conducted in the absence of any commercial or financial relationships that could be construed as a potential conflict of interest.
